# Gene expression profiles in mouse embryo fibroblasts lacking stathmin, a microtubule regulatory protein, reveal changes in the expression of genes contributing to cell motility

**DOI:** 10.1186/1471-2164-10-343

**Published:** 2009-07-30

**Authors:** Danielle N Ringhoff, Lynne Cassimeris

**Affiliations:** 1 Chemistry Department, Lehigh University, Mudd Building, 6 E. Packer Avenue, Bethlehem, PA, USA; 2 Department of Biological Sciences, Lehigh University, Iacocca Hall, 111 Research Drive, Bethlehem, PA 18015, USA

## Abstract

**Background:**

Stathmin (STMN1) protein functions to regulate assembly of the microtubule cytoskeleton by destabilizing microtubule polymers. Stathmin over-expression has been correlated with cancer stage progression, while stathmin depletion leads to death of some cancer cell lines in culture. In contrast, stathmin-null mice are viable with minor axonopathies and loss of innate fear response. Several stathmin binding partners, in addition to tubulin, have been shown to affect cell motility in culture. To expand our understanding of stathmin function in normal cells, we compared gene expression profiles, measured by microarray and qRT-PCR, of mouse embryo fibroblasts isolated from STMN1^+/+ ^and STMN1^-/- ^mice to determine the transcriptome level changes present in the genetic knock-out of stathmin.

**Results:**

Microarray analysis of STMN1 loss at a fold change threshold of ≥ 2.0 revealed expression changes for 437 genes, of which 269 were up-regulated and 168 were down-regulated. Microarray data and qRT-PCR analysis of mRNA expression demonstrated changes in the message levels for STMN4, encoding RB3, a protein related to stathmin, and in alterations to many tubulin isotype mRNAs. KEGG Pathway analysis of the microarray data indicated changes to cell motility-related genes, and qRT-PCR plates specific for focal adhesion and ECM proteins generally confirmed the microarray data. Several microtubule assembly regulators and motors were also differentially regulated in STMN1^-/- ^cells, but these changes should not compensate for loss of stathmin.

**Conclusion:**

Approximately 50% of genes up or down regulated (at a fold change of ≥ 2) in STMN1^-/- ^mouse embryo fibroblasts function broadly in cell adhesion and motility. These results support models indicating a role for stathmin in regulating cell locomotion, but also suggest that this functional activity may involve changes to the cohort of proteins expressed in the cell, rather than as a direct consequence of stathmin-dependent regulation of the microtubule cytoskeleton.

## Background

Stathmin (STMN1) is a ubiquitous microtubule (MT) destabilizing protein linked to cancer and cell health: Stathmin is highly over-expressed in leukemias and a number of other cancers, where its expression level often correlates with cancer stage progression and prognosis for survival [1-3]. Stathmin is the founding member of a family of MT destabilizers known as the stathmin family of proteins, which includes SCG10 (STMN2), SCLIP (STMN3), and RB3 (STMN4) [4-7], each expressed from separate genes. Each of the four stathmin family proteins shares a homologous tubulin binding site that functions as both a MT destabilizer and tubulin heterodimer sequestering protein. Stathmin is expressed in a wide range of tissues and is present as a soluble cytosolic protein [8], while SCG10 (Superior cervical ganglion-10 protein; [6,7,9], SCLIP (SCG10 like protein, [5]), and RB3 (with splice variants RB3'/RB3", stathmin-like protein B3; [4,7]) are neuron-specific homologues of stathmin localized to membranes in developing (SCG10 and SCLIP) and mature (RB3/RB3'/RB3") nerve cells.

Though it is well established that stathmin regulates MTs, many have suggested alternative functions for stathmin. Stathmin has been called a cell survival factor because its level of overexpression correlates with cancer stage progression, invasion, and metastasis for many cancer types (reviewed by [1]). For example, knockdown of stathmin protein by siRNA [10], shRNA [11-13], or ribozymes [14] leads to apoptosis of several cancer cell lines in culture.

Stathmin has also been linked to cell motility and metastasis. Overexpression of stathmin stimulates motility of both GN-11 neurons [15] and HT-1080 fibrosarcoma cells [16,17]. In fibrosarcoma cells, stathmin activity is regulated by p27^kip1 ^[16]. Ng and coauthors [18] have also proposed a role for stathmin in mouse embryonic fibroblast (MEF) cell migration, although their results indicate that stathmin inhibits, rather than promotes, cell migration. In this study, stathmin activity was regulated by the transcription factor STAT3 [18]. The ability of stathmin to positively or negatively regulate motility may be context-specific, where stathmin promotes motility in 3D matrices, but not in 2D [17].

Although stathmin has a significant function in regulating the MT cytoskeleton, surprisingly STMN1 knockout (STMN1^-/-^) mice develop normally except for some minor age-onset axonopathies associated with STMN1^-/- ^[19] and a lack of learned or innate fear response [20]. It is not known why stathmin is dispensable for normal development but required for survival of many cancer cell lines. For example, it is not known whether compensatory changes occur in mice lacking STMN1 to permit normal development.

Here we performed an unbiased screen for transcriptome level changes associated with genetic knockout of STMN1. Microarray studies of MEFs, genetically designated STMN1^+/+ ^or STMN1^-/-^, were used to compare global transcriptome level changes due to deletion of the STMN1 gene. Examination of genes encoding proteins either related to stathmin, or regulated by stathmin, revealed differential expression of the stathmin family proteins and tubulin isotypes. Additional classification of differential regulation was performed using Gene Ontology (GO) and KEGG pathway analyses, and demonstrated that expression of genes functioning in cell adhesion and extracellular matrix make up the majority of up and down regulated genes, supporting a role for STMN1 in cell motility.

## Methods

### Isolation of MEFs

C57BL/6 STMN1^+/- ^male and female mice (gift of G. Shumyatsky, Rutgers University) were mated. 13.5 days to 14.5 days post coitus, pregnant females were sacrificed by cervical dislocation, and fibroblasts were isolated as described by Tessarollo [21]. Fibroblast cells from individual embryos were plated and allowed to grow for 1–3 days prior to storage of aliquots in liquid nitrogen.

**Genotyping **of MEFs was performed as described by Liedtke [19]. Briefly, DNA was isolated from embryonic tissue using isoamyl alcohol/phenol extraction methods. Samples were amplified using PCR to identify embryos with intact STMN1 intron III or the neomycin cassette used to disrupt the STMN1 gene [22]. The PCR Jump Start^® ^REDTaq Kit (Sigma-Aldrich) was used for amplification; each sample contained 1.5 mM MgCl_2_, deoxynucleotide triphosphates (200 μM each), primers (0.5 μM each), and Taq polymerase (0.05 U/μL). For the wild-type amplification, 35 temperature cycles were performed (95°C, 35 seconds; 66°C, 45 seconds; 72°C, 45 seconds). The following primer set was used as a probe for wild-type alleles: forward sense primer 5'-3' (GAGAATCCATGATTGCCAGC), corresponding to a region of intron III deleted in the mutant allele; and a reverse anti-sense primer 5'-3' (AGAAACCAGTAGAGGGCATCA) also missing in the mutant, corresponding to a region of intron III of the STMN1 gene that yields an amplification product 317 bp long. A second set of reactions were performed using mutant-allele specific primers that anneal to the neomycin insert [22]; forward sense primer 5'-3' (CTTGGGTGGAGAGGCTATTC) and a reverse anti-sense primer 5'-3' (AGGTGAGATGACAGGAGATC), corresponding to a region of the inserted neomycin cassette of the mutant allele that yields an amplification product 280 bp long.

### Cell Culture

All cells were cultured at 37°C in a humidified atmosphere of 5% carbon dioxide. Cells were grown in DMEM (pH 7.4) supplemented with 4.5 g/L D-glucose, L-glutamine (GIBCO-Invitrogen), 44 mM sodium bicarbonate, 1× antibiotic/antimycotic (Sigma-Aldrich, St. Louis, MO), 1 mM sodium pyruvate (Sigma-Aldrich), and 10% fetal bovine serum (FBS) (Invitrogen). Cell cultures were passaged at ~80% confluency, about every 3–4 days. Cultures were discarded after passage 7.

### RNA Isolation

Total RNA was isolated from cultured MEF cell lines using TRIzol^® ^Reagent (Invitrogen, Carlsbad, CA) following manufacturer's instructions, followed by DNase treatment. RNA quality was assessed by A260/A280 ratio (Nanodrop) and further validated by the Agilent 2100 Bioanalyzer. The same mRNA was used in both microarray and qRT-PCR experiments.

### Microarray

STMN^+/+ ^and STMN1^-/- ^mRNA were fluorescently labeled with either Cy3 or Cy5 using the Agilent Low RNA Input Fluorescent Linear Amplification Kit (Agilent). Hybridized two-color samples were prepared using Agilent Whole Mouse Genome 4 × 44 k Oligo Microarray Kit format (G4122F) according to Agilent instructions and using Agilent reagents. Each array includes 43,379 biological features with a total of 45,018 probes of 60-mer controls and gene probes. Arrays were run as color-swap duplicates and scanned with an Agilent Microarray Scanner System, which generated the TIFF images of low and high intensity scans utilized by Agilent Feature Extraction Software (v9.5). Feature Extraction processing of fluorescent data corrected signals for background noise, foreground intensities, positive and negative spot controls, background subtraction, and signal normalization. Results were collected into tab delimited text files for each of the four experimental arrays for analysis with Agilent Technologies software GeneSpring GX (v10.0.1). Data were processed in GeneSpring GX (v10.0.1) by first filtering on flags for features either present or marginal in any 1 of 4 arrays, followed by error filtering to a coefficient of variance < 50%, then statistical analysis using the T-test against zero with a false discovery rate of < 0.05. The resultant list comprised 7090 genes, 3510 of which were up-regulated and 3580 down-regulated. Further analyses were done using fold change cut-offs.

### qRT-PCR

RNA was used to synthesize cDNA with SuperScript™ III First-Strand Synthesis System for quantitative RT-PCR (qRT-PCR; Invitrogen). RNA was isolated from at least two cell lines from each genotype for cDNA preparation and qRT-PCR. All stathmin family proteins and isotypes of mouse α- and β-tubulin were probed using primers listed in [Additional file 1]. Oligonucleotide probes were designed using Primer Express^® ^Software (Applied Biosystems, Foster City, CA) [see Additional file 1] and cDNA sequences from the mouse genome [23]. For each probe, we used at least three dilutions of cDNA, each dilution run in triplicate. Commercial 96-well plate arrays for the focal adhesion and extracellular matrix pathways were also assayed for each MEF genotype (SABiosciences, Frederick, MD). Two plates were run for each genotype. Message levels were quantified for each genotype using oligonucleotide probes designed to amplify cDNA fragments containing target gene exons. Results were normalized to the signal from GAPDH for designed probes, or to the signal from five internal standards (Gusb, Hprt1, Hsp90ab1, Gapdh, and Actb) for the SABiosciences arrays.

Amplification was achieved using Power SYBR^® ^Green PCR Master Mix (Applied Biosystems) and Applied Biosystems 7300 Real Time PCR System with SDS v1.4 Software. RT-PCR amplicon specificity was checked by electrophoresis of RT-PCR products on 2% agarose gels (data not shown). Following manufacturer instructions (Applied Biosystems), the threshold for determining C_T _values was set to Log scale 0.2 and internal normalization of genotype results to GAPDH was first calculated (i.e., C_T _Target mRNA-C_T _GAPDH mRNA = ΔC_T_). The C_T _values for GAPDH were stable and consistent across genotypes. One measure of the consistent GAPDH amplification is the small standard deviations for the GAPDH C_T _among replicates. The standard deviation for GAPDH was ≤ 0.1 – 0.4% of the mean C_T _value. ΔC_T _values were not calculated for target mRNA samples that exceeded 35 cycles prior to crossing the threshold. The relative abundance of target mRNA between genotypes was calculated as 2 raised to the negative of the difference in ΔC_T _values



All statistical analyses were done using EXCEL (Microsoft Corporation 2007). RT-PCR data are presented as fold changes relative to MEF STMN1^+/+ ^samples. Standard errors (SE) were computed using ΔC_T _values transformed to SE values of fold change using the formula:



Significance was determined by ANOVA of ΔC_T _values [24,25]. Amplification efficiency was determined to be 100% for GAPDH for each genotype per manufacturer's instructions (data not shown), and it was assumed that efficiency of all target genes was also 100% for our statistical analysis.

## Results

### Microarray Analysis of Gene Regulation in STMN1^-/- ^MEFs

**Figure 1:**
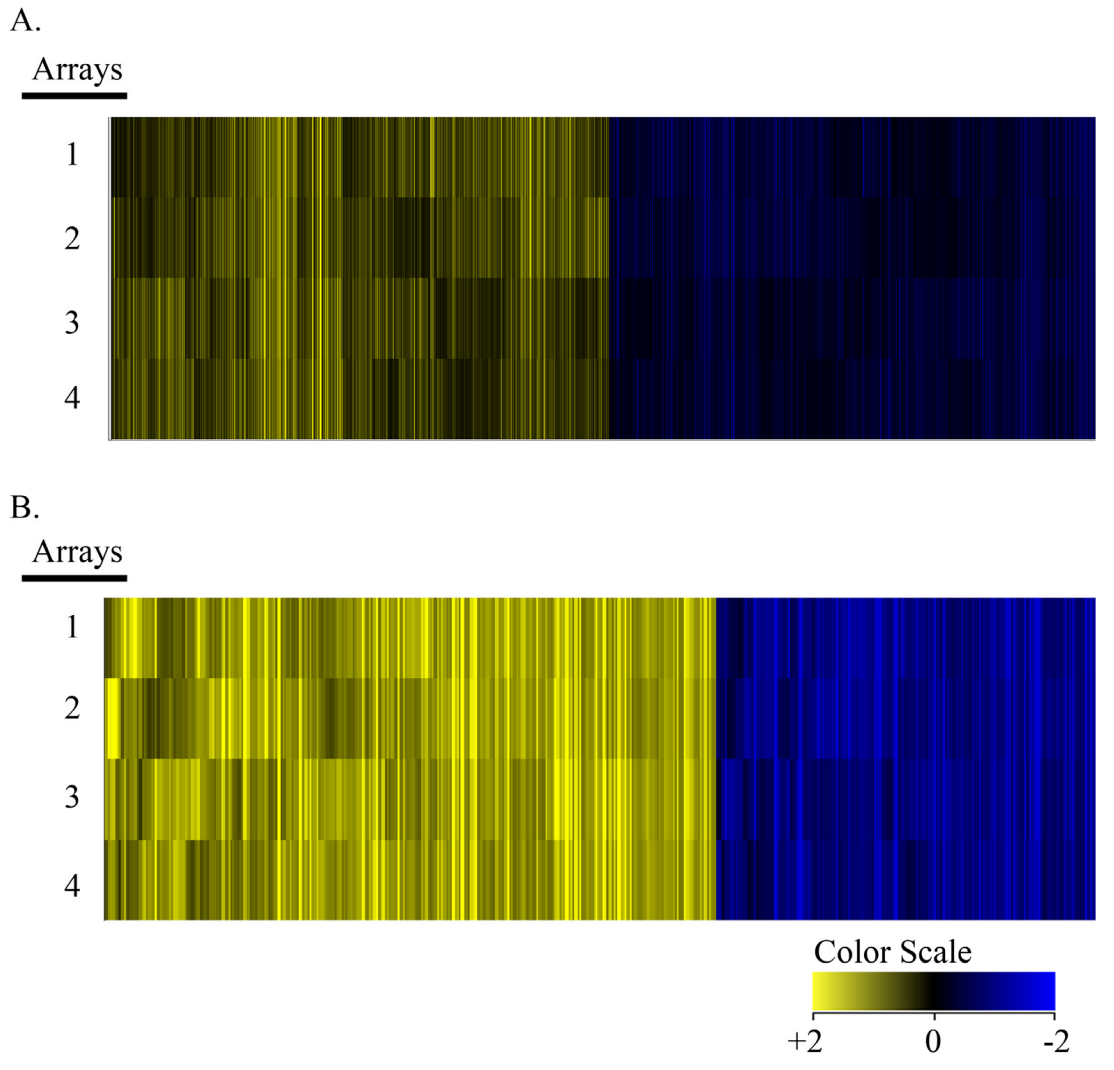
**STMN1^-/- ^differentially expressed genes on 2-color microarrays illustrated in heatmaps**. (A) Heatmap showing 3953 transcripts filtered with a fold change threshold ≥ 1.2, showing 1997 up-regulated and 1956 down-regulated genes relative to stmn1^+/+^. (B) 437 transcripts filtered to a fold change level ≥ 2, showing 269 up-regulated and 168 down-regulated genes. Numbers 1 – 4 refer to replicates. Color scale bar indicates up-regulation in yellow and down-regulation in blue.

Changes to stathmin protein levels regulate microtubule polymer in MEFs [18,26] and cancer cell metastatic potential and invasiveness [15-18]. To probe whether STMN1 gene knockout resulted in a change to the MEF gene expression profile, we identified the transcriptome differences between STMN1^+/+ ^and STMN1^-/- ^MEFs using Agilent 2-color microarrays run in quadruplicate with Cy3/Cy5 dye color-swap. The initial 7090 statistically significant probes were further parsed for differential gene expression fold change of ≥ 1.2 to encompass as many potentially relevant gene-level alterations as possible. The inclusive list of genes totaled 5991 probes, of which 3011 were up-regulated and 2980 were down-regulated in STMN1^-/- ^compared to the STMN1^+/+ ^controls. Of the 5991 probes, 3953 have known functions. Of these, 1997 were up-regulated and 1956 were down-regulated as shown in the heatmap of Figure 1A. More stringent analysis was performed on the list of 3953 genes at a fold change threshold of ≥ 2.0. At this level, comparison of STMN1^+/+ ^and STMN1^-/- ^cell lines resulted in expression changes for 437 genes [see Additional file 2], of which 269 were up-regulated and 168 were down-regulated (Figure 1B).

### Expression of stathmin family and tubulin isotypes measured by microarray

Stathmin is the founding member of a family of proteins, all of which contain a homologous tubulin binding site [27,28]. The microarray did not show complete loss of STMN1 in the STMN^-/- ^MEFs because the probe design for STMN1 is flawed. The Agilent 60-mer was found to match both STMN1 on chromosome 4 and a region of chromosome 9, a source of immunodominant MHC-associated peptides (SIMP) [29,30]. However, by both qRT-PCR and western blotting, STMN1 mRNA and protein are absent in STMN^-/- ^MEFs [26]. We next looked for changes to stathmin family mRNAs as a consequence of STMN1 knockout and found that STMN2 decreased by 2.4 fold and STMN4 increased by 2.3 fold (Table 1). STMN3 was not found to be differentially regulated on the microarray. Tubulins are expressed from 8 α-tubulin genes and 8 β-tubulin genes. The cohort of tubulin isotypes present can assemble into MTs with different dynamic properties in vitro [31]. Therefore, we examined whether STMN1 genotype influences the tubulin isotype expression profile of each cell line. No significant changes in expression of α-tubulin isotypes were detected. In contrast, small, but significant (FDR < 0.05) changes in the expression levels of β-tubulin isotypes were observed in the microarray (Table 1), with β-tubulin isotype Classes IIa/b increased 1.4/1.5 fold, Class-III increased 1.2 fold, Class IVb decreased 1.4 fold, and Class-V decreased 1.3 fold.

**Table 1:** Microarray and qRT-PCR comparison

	**Microarray Results**	**qRT-PCR Results**	
**MEF STMN1^-/-^**			

STMN2	-2.44	1.27	
STMN3	N/A	N/A	
STMN4	2.26	8.06	*
Tubb2a/Class IIa	1.40	1.80	*
Tubb2b/Class IIb	1.54	2.64	*
Tubb2c/Class IVb	-1.35	-0.57	*
Tubb3/Class III	1.22	1.78	*
Tubb6/Class V	-1.31	-0.57	*

Magnitude and direction of gene expression changes found using microarray analysis compared to qRT-PCR with designed primers. Only those tubulin isotypes whose expression was significantly changed, as measured by microarray with FDR < 0.05, are shown compared to qRT-PCR (*, p-value < 0.005). Data are given as fold change in STMN^-/- ^MEFs relative to STMN^+/+ ^MEF.

### Confirmation of stathmin family and tubulin isotype expression by qRT-PCR

Changes to stathmin family mRNA and tubulin isotype levels were verified by qRT-PCR using designed primers [see Additional file 1]. For STMN1^-/- ^cells relative to STMN^+/+ ^cells, upregulation of STMN4 (RB3) was seen by both microarray and qRT-PCR. In contrast, STMN2 (SCG10) showed down regulation by microarray, but upregulation by qRT-PCR (Table 1). The reason for the difference in expression pattern by the two methods is not known but suggests that changes in STMN2 expression are either not significant or not consistently altered compared to the level in WT cells. We also used qRT-PCR to examine the message levels for stathmin and related proteins in MEFs heterozygous for stathmin (STMN^+/-^). The STMN1^+/- ^cells showed a 1.8 fold increase in STMN2, similar to the increased expression in STMN^-/- ^MEFs measured by qRT-PCR. For STMN4, mRNA was increased 3 fold in STMN^+/- ^MEFs compared to that in wild-type cells, consistent with the up regulation observed in STMN^-/- ^cells. The STMN3 mRNA was not present at quantifiable levels in RT-PCR for any of the three genotypes, consistent with the microarray for wild-type and knockout genotypes. Similar changes in stathmin family expression were also observed in an additional cell line for each genotype. Protein products other than stathmin were undetectable with available antibodies, suggesting that protein products of STMN2 and STMN4 are present at low levels.

**Figure 2:**
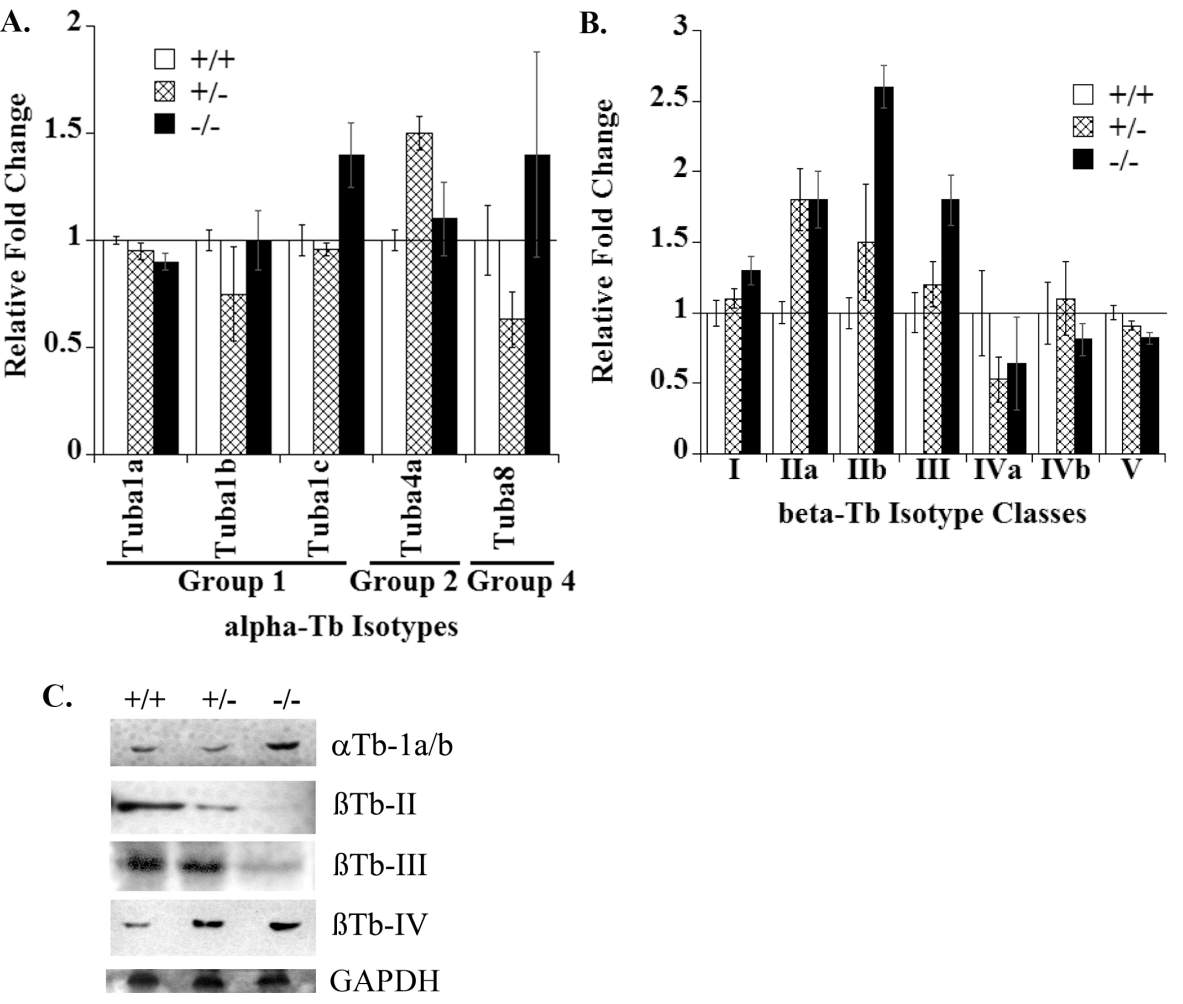
**Tubulin isotypes are differentially regulated with STMN1 knock-out**. Tubulin isotypes for α- and β-tubulin mRNAs were measured by qRT-PCR (Methods). (A) α-tubulins and (B) β-tubulins. (C) Protein levels were measure by immunoblots of whole cell lysates from each genotype, loading control is GAPDH.

qRT-PCR was also used to confirm expression of tubulin isotypes for each mouse tubulin isotype. MEFs expressed five α- and seven β-tubulin isotypes at quantifiable levels by qRT-PCR (Figure 2). For the α-tubulin isotypes, STMN^-/- ^MEFs expressed slightly higher levels of Tuba1c and Tuba8 mRNAs compared to wild-type MEFs (Figure 2A) [see Additional file 3 for isotype nomenclature]. By microarray, the probes for Tuba1c and Tuba8 did not pass the stringency filters applied to the raw data set and were not flagged as significantly changed in expression. Examination of the unfiltered microarray data showed opposite regulation to that measured by RT-PCR for Tuba1c and Tuba8. The discrepancies between RT-PCR and microarray may reflect difficulty detecting small changes in expression level. As shown in Table 1, microarray and RT-PCR detected similar changes in expression of five β tubulins isotypes. STMN^-/- ^MEFs showed higher expression of isotype classes IIa, IIb and III, while isotype classes IVb and V were reduced in STMN1^-/- ^MEFs relative to wild-type cells (Figure 2B, Table 1). By RT-PCR, we also detected upregulation of β-tubulin class I and down regulation of class IVa in STMN^-/- ^MEFs compared to wild-type cells (Figure 2B). The microarray data for class I and IVa β-tubulins did not pass the applied data filters. Although they did not pass the data filters, class IVa tubulin showed reduced expression by both RT-PCR and microarray, but changes to class I tubulin were in opposite directions by microarray and RT-PCR. Therefore, with a few exceptions, the data from RT-PCR for tubulin isotypes generally confirmed the microarray results. Microarray results for STMN 3 and STMN4 were also confirmed by qRT-PCR, and in general, both STMN family and tubulin isotype expression confirmed the microarray results (Table 1). Correlation between microarray and qRT-PCR can depend on a large number of factors [32]. Qualitatively, the correlations we found between microarray and qRT-PCR are consistent with those described by others [32].

In addition, we also examined tubulin isotype expression in the heterozygous STMN1^+/- ^cell line and observed changes in β-tubulin mRNA levels similar to that measured in knockout MEFs. We also found that the STMN1^+/- ^cell lines expressed lower mRNA levels of most α-isotypes, but showed higher expression of Tuba4a mRNA compared to wild-type cells.

Tubulin mRNA levels do not always correlate with measured protein levels [33,34]. Therefore, we used Western blots to estimate relative protein levels for those isotypes where specific antibodies are available [see Additional file 3]. The anti-α tubulin antibody DM1A recognizes the C-terminal amino acid sequence found in Tuba1a and Tuba1b. Immunoblots probed with DM1A indicated that the combined protein level of Tuba1a and Tuba1b was reduced in heterozygous cells, which is consistent with the decreased mRNA level for each isotype relative to wild-type cells. The combined protein level of Tuba1a and b was higher in STMN1^-/- ^cells compared to wild-type cells, while the corresponding mRNA levels were slightly reduced in the STMN1^-/- ^cells (Figure 2C). The protein levels for β-tubulin isotype classes II and III decreased with loss of STMN1, while class IV β-tubulin increased in the STMN1^-/- ^line. For each MEF cell line, the protein levels of β-tubulin isotypes were inversely related to their corresponding mRNA level. For example, in STMN1^-/- ^cells, both β-tubulin classes II and III showed increased mRNA levels and decreased protein levels relative to wild-type cells. For class IV β-tubulins, mRNAs decreased while protein level increased in STMN1^-/- ^cells relative to wild-type cells.

### STMN1 genotype and expression of MT regulators and motors

The microarray data were then examined for expression of MT regulators and motor proteins, the kinesin family and dyneins, which transport cargo along MTs. As shown in Table 2, we found that STMN1 knockout resulted in small fold-change differences to a number of MT stabilizing and destabilizing proteins, without a clear pattern of up-regulation of expression for proteins that might compensate for stathmin or for down-regulation of proteins that normally work in opposition to stathmin. Most notably, we did not see a change in the expression of MAP4, a protein previously shown to function antagonistically to stathmin [35-37]. In general, expression of genes for other proteins that destabilize MTs, including MT severing proteins, were down-regulated in STMN^-/- ^MEFs relative to STMN1^+/+ ^cells, making it unlikely that these MT destabilizers substitute for STMN1. Surprisingly, the largest group of genes whose expression in STMN1^-/- ^MEFs differed from that in WT MEFs was the group encoding motor proteins. We found differential expression of 13 kinesin genes and 5 genes encoding dynein light or intermediate chains. Two kinesins, Kif 21b and Kif26b showed > 3 fold up-regulation in STMN1^-/- ^MEFs. These two kinesins have not been well studied and it is unclear what the consequences of their up regulation may be.

**Table 2:** MT Regulators and Associated Protein Profiles

**Functional Class**	**Common Name**	**Gene Name**	**Fold Change**
***MT Polymerization Regulators***			

	EB2	mapre2	1.20
	MAP1A	mtap1	1.47
	MAP1B	mtap1b	1.27
	MAP2	mtap2	-1.63
*Stabilizers/Assembly Promoters*	MAP4	mtap4	1.24
	CLASP1	Clasp1	-1.19
	MACF2/BPAG1	Dst	-1.29
	STOP	mtap6	-1.16
	YB-1	Ybx1	-1.24
	Dia2	Diap2	-1.19

	RHAMM	Hmmr	-1.57
*Mitosis-specific stabilizers*	TACC3	Tacc3	-1.17
	TPX2	Tpx2	-1.29

	SCG10	stmn2	-2.44
*Destabilizers/Disassembly Promoters*	RB3	stmn4	2.26
	Kif2A	Kif2a	-1.22
	Kif18A	Kif18a	-1.62

***MT Severing Proteins***			

	Katanin p60	katnal2	-1.37
	Fidgetin	Fign	-1.92

***MT Nucleators***			

	gamma-tubulin 2	Tubg2	1.27
	GCP3	Tubgcp3	-1.18

***MT-based Motors***			

	Kif11	Kif11	-1.24
	Kif15	Kif15	-1.45
	Kif18a	Kif18a	-1.62
	Kif1a	Kif1a	1.77
	Kif20a	Kif20a	-1.15
	Kif21a	Kif21a	-1.74
*Kinesins*	Kif21b	Kif21b	4.60
	Kif22	Kif22	-1.19
	Kif26a	Kif26a	2.32
	Kif26b	Kif26b	3.24
	Kif2a	Kif2a	-1.22
	Kif4	Kif4	-1.27
	Kif9	Kif9	1.15

	dynein axonemal LC4	Dnalc4	1.26
	cytoplasmic dynein 1 IC1	Dync1i1	-1.29
*Dyneins*	cytoplasmic dynein 1 LIC1	Dync1li1	-1.18
	cytoplasmic dynein 1 LIC2	Dync1li2	1.20
	dynein LC8-type2	Dynll2	-1.18

Magnitude and direction of gene expression changes found using microarray analysis. Gene names are in *M. musculus *format, fold changes are from microarray data.

### STMN1 loss impact categorized by KEGG Pathways and Gene Ontology

The analyses described above involved directed searches for genes encoding proteins functioning as part of the MT cytoskeleton. To expand our analysis and take advantage of the non-biased nature of microarrays, we performed pathway analysis on those genes showing a ≥ 2.0 fold change using the online resource KEGG Pathways (KEGG, Kyoto Encyclopedia of Genes & Genomes online resource, )[38] for *Mus musculus*. Top pathway hits include Focal Adhesion (18 genes), Extra-Cellular Matrix (ECM) Receptor Interactions (15 genes), Cell Communication (15 genes), and Regulation of Actin Cytoskeleton (12 genes) [see Additional file 4].

**Figure 3:**
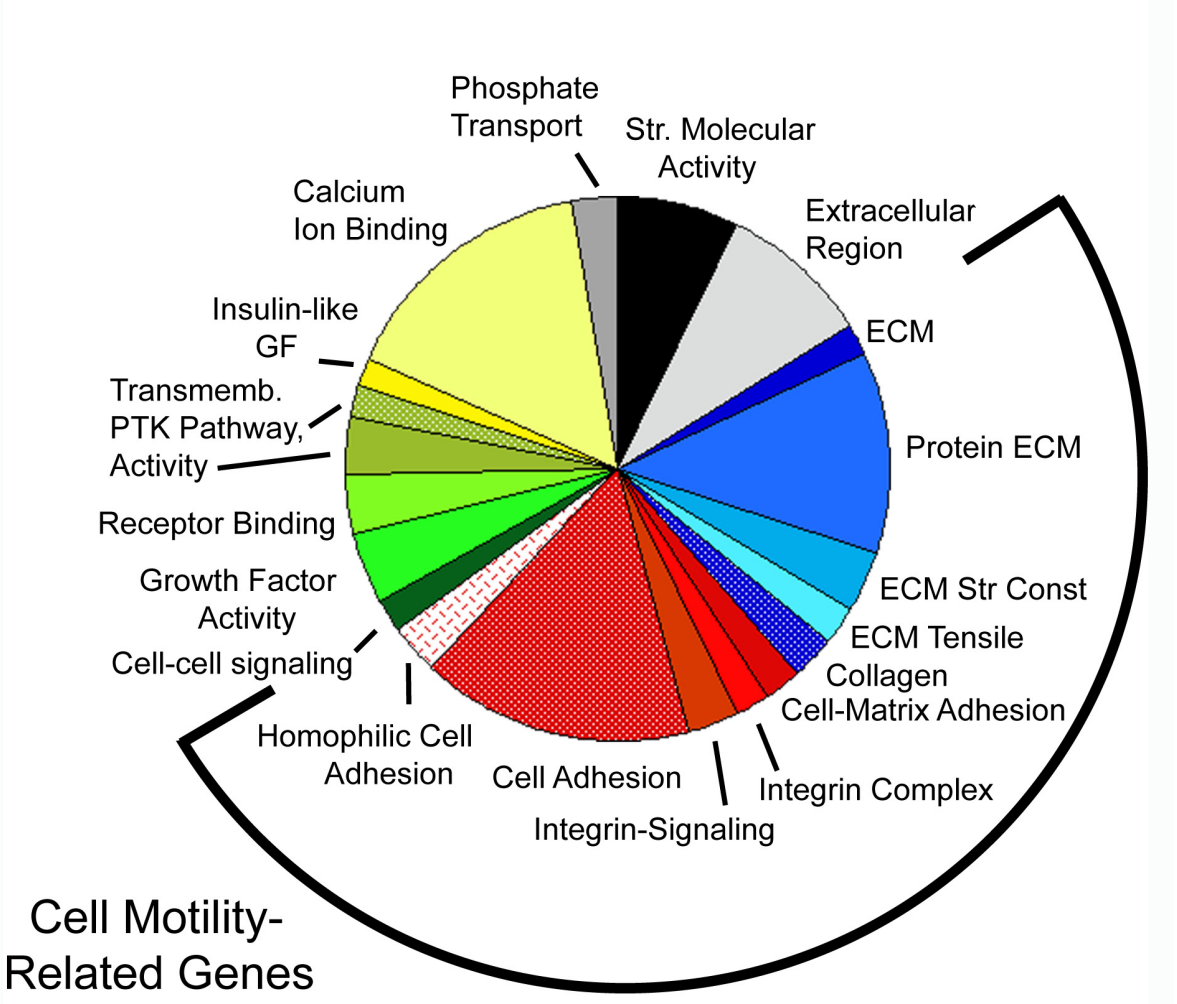
**GO terms of microarray genes differentially regulated by ≥ 2-fold with p-values < 0.5 using KEGG Spider**. Expression data are from microarray analysis of transcripts showing ≥ 2-fold expression change with p-values < 0.5 and grouped into GO terms using KEGG Spider. The pie chart illustrates the ECM-related proteins in shades of blue, adhesion-related proteins in shades of red, signaling-factors in shades of green, and others in shades of gray. 50% of all GO terms identified can be broadly grouped into cell motility-related genes. For a list of differentially expressed genes and corresponding GO Terms, see [Additional file 5].

An alternate tool to KEGG Pathway analysis is the newly developed KEGG Spider [39]. KEGG Spider was used to better characterize the interconnectedness of the differentially expressed genes. The output from KEGG Spider includes enriched Gene Ontology (GO) categories based on an interconnection network algorithm that also provides p-value significance measures. GO characterization of 437 genes with fold changes ≥ 2.0 was performed within KEGG Spider with a p-value cutoff of 0.5, yielding 27 individual categories [see Additional file 5]. The most highly represented GO terms included cell adhesion and extracellular matrix, and the sum of all cell motility-related genes comprised about 50% of all GO terms represented (Figure 3).

**Figure 4:**
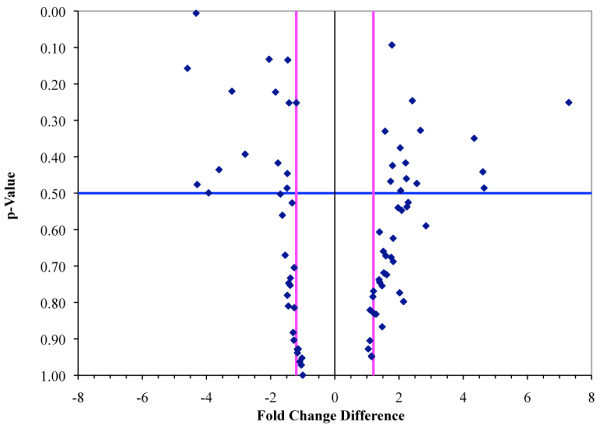
**Expression changes in STMN^-/- ^MEFs for genes encoding focal adhesion and ECM related pathways measured by qRT-PCR**. The fold change threshold of mRNAs representing focal adhesion & ECM related genes in STMN^-/- ^versus STMN1^+/+ ^was set to ≥1.2 (vertical pink lines) with p-value of 0.5 (horizontal blue line). All genes falling into either the upper left (significantly down regulated) or upper right (significantly up regulated) quadrants were used for comparison to microarray data.

Given the changes to genes implicated in cell adhesion and cell motility, we used a pathway specific (Mouse Extracellular Matrix and Adhesion Molecules PCR Array) RT-PCR kit to further confirm the microarray results. Relative transcript levels in STMN1^+/+ ^and STMN1^-/- ^MEFs were measured for 84 focal adhesion and ECM mRNAs. Data that met a fold change threshold of ≥ 1.2 and p-value of < 0.5 in the PCR array were considered significant for comparison to microarray data (Figure 4). There were 15 differentially regulated genes that matched in the direction (up or down) of fold change, while 6 genes were oppositely regulated compared to the microarray (Table 3). The majority (71%) of mRNAs for cell adhesion and ECM were up or down regulated in the same direction (up or down) for both microarray and qRT-PCR, providing general support for the microarray results. The most extreme differentially expressed entities in STMN1^-/- ^compared to STMN1^+/+ ^measured by the qRT-PCR array were catenin β 1 (Ctnnb1) which was down-regulated 4.6 fold in support of enhanced migration, and P-Selectin (Selp) which was up-regulated by 7.3 fold.

**Table 3:** Comparison of microarray and qRT-PCR measurements for genes encoding focal adhesion and ECM components

**Symbol**	**Microarray**	**RT-PCR Array**
Ctnnb1	-0.90	-4.59
Itgb2	-1.10	-4.32
Spock1	-0.89	-3.61
Adamts1	-0.47	-3.21
Mmp10	-1.27	-2.80
Itgav	-1.33	-1.85
Entpd1	-1.45	-1.77
Itga3	-2.05	-1.47
Itgb4	-1.69	-1.20
Ctnna1	1.96	1.78
Mmp11	1.95	2.04
Col6a1	1.92	2.21
Adamts2	3.34	2.67
Cdh3	1.62	4.34
Selp	1.65	7.29
Thbs1	1.26	-2.05
Postn	3.61	-1.43
Mmp14	-0.66	1.56
Sgce	-0.92	1.80
Cdh4	-1.36	2.42
Adamts5	-1.33	4.61

The qRT-PCR measurements were made from a commercial 96-well plate format. Data for qRT-PCR is shown as the mean of duplicate assays. The top 15 entries show mRNAs with matching directionality of expression fold change between RT-PCR and microarray. The last 6 entries list MRNAs in which expression fold change measured by RT-PCR is in the opposite direction of the microarray data.

## Discussion

Stathmin is an integral protein involved in control of MT polymer level during interphase of the cell cycle [26,37,40]. Many cancers express high levels of stathmin (e.g. [1]) and increased stathmin level is correlated with reduced patient survival [2,3,10,41,42]. Cancer cells over-expressing stathmin protein display invasive and metastatic behaviors [17], and alternative stathmin binding partners have recently been revealed [16,18]. These roles for stathmin in regulation of the MT cytoskeleton and cell motility, and stathmin's as yet undefined role(s) in cancer, led us to use microarray technologies as an unbiased screen for transcriptome level changes due to genetic knockout of the STMN1 gene to provide clues to stathmin function beyond the MT cytoskeleton.

### Loss of STMN1 is Correlated with Expression Changes to Genes Encoding Proteins Functioning in Cell Motility Pathways

Several recent studies have indicated a role for stathmin in regulation of cell motility [15-18]. Here, we show that genetic knockout of STMN1 has transcriptome level impact on genes broadly classified in cell motility pathways, including integrins, ECM components, and cell adhesion (Figure 3), which comprised 50% of all GO categories derived from our microarray data. Additionally, qRT-PCR using a pathway specific array gave strong supporting evidence of cell adhesion and ECM protein transcript changes as a result of STMN1 loss (Table 3). Of the 15 differentially regulated genes that match results from the 4 microarray replicates, 4 integrins are represented, thus linking changes in STMN1 expression to change in focal adhesion proteins that also link to the actin cytoskeleton. Although we cannot predict whether the measured changes in gene expression would promote or inhibit cell migration, our data point to a role for stathmin in regulating the expression level of a wide range of proteins required for cell adhesion and locomotion. These data indicate that STMN1 knockout has consequences beyond simply regulating assembly of the MT cytoskeleton by regulating the expression level of a cohort of proteins required for cell motility. Whether these expression changes are a downstream consequence of altered MT assembly dynamics is not known.

### Loss of STMN1 impacts the MT system

Cells differing in STMN1 genotype expressed different mRNA levels for the stathmin related protein STMN4 (RB3), with STMN^-/- ^MEFs showing upregulation of STMN4, by either microarray or RT-PCR analysis. We cannot detect STMN4 expression in MEFs with antibodies currently available, indicating that STMN4 protein is likely present at a low level in these cells. We note that the expression of other MT destabilizers are reduced in STMN1^-/- ^MEFs, making it unlikely that cells compensate for loss of STMN1 through expression of STMN4 or other MT destabilizers (Table 2).

Several previous reports have also noted that stathmin family proteins are upregulated in STMN1 knockout mice. Liedtke *et al*. [19] demonstrated that STMN3 expression is higher in the brains of aged knock-out mice and Yoshie *et al*. [43] showed increased expression of STMN3 in the peri-implantation uteri of knockout mice. It is possible that STMN3 may compensate for loss of STMN1 in these tissues. We did not detect changes in STMN3 expression in STMN1^-/- ^MEFs, either by microarray or qRT-PCR, indicating that STMN3 does not compensate for STMN1 in these cells.

In addition to changing expression levels of STMN4, STMN1^-/- ^MEFs also expressed different levels of tubulin isotypes compared to wild-type cells (Figure 2). Tubulin isotype mRNA levels do not necessarily correspond to protein levels [33,34], as we also found in MEFs. For example, STMN1^-/- ^MEFs expressed higher levels of β-tubulin class III mRNA but lower levels of the corresponding protein (Figure 2). Alterations to β-tubulin protein isotype ratio, specifically the levels of classes II, III, and IV proteins, can impact rates of MT growth and shortening in vitro [31,44] although this is not always observed in cells [45]. Changes to β-tubulin isotype protein expression have also been linked to paclitaxel resistance in cancers [45,46] via increased MT dynamic instability. In MEFs, the amount of β-tubulin class II and III proteins fell dramatically with loss of STMN1 while class IV protein increased. Class II and IV form more stable MTs, while class III forms more dynamic MTs in vitro [31], and therefore, MEFs do not display a clear pattern of tubulin isotype up or down regulation to compensate for loss of stathmin.

## Conclusion

Our microarray and qRT-PCR results showed that deletion of both copies of the STMN1 gene resulted in changes to the expression level of many genes, including a large cohort of genes related to cell adhesion and motility. It is possible that stathmin depletion has additional, or different, consequences in those cells where it is expressed at higher levels than that in embryonic fibroblasts. Overall, our data support a role for STMN1 in cell migration and cross-talk between the MT and actin components of the cytoskeleton [47], as suggested by Baldassarre et al. [16] and Belletti et al. [17].

## Authors' contributions

DNR participated in experimental design, performed experiments and analyses; assembled tables and figures and drafted the manuscript. LC participated in experimental design, assembly of tables and figures and helped to draft the manuscript. All authors read and approved the final version of the manuscript.

## Authors' information

DNR is a graduate student in the Chemistry Department, Lehigh University. The manuscript describes a portion of her Ph.D. research project in LC's laboratory in Biological Sciences, Lehigh University.

## Supplementary Material

Additional file 1 **Primers designed for stathmin family and tubulin isotype qRT-PCR**. All primers were designed as described in Methods. Bold nucleotides represent exon junctions in mRNAs. Click here for file

Additional file 2 **Genes differentially expressed in STMN1^-/- ^MEFs compared to STMN1^+/+ ^MEFs**. Differentially expressed genes listed with accession numbers, gene symbols, and average fold change over quadruplicate microarrays with color-swap. Click here for file

Additional file 3 **Tubulin isotype mouse versus human nomenclature and antibody specificities**. Tubulin isotypes for α- and β-tubulins are listed with accession numbers, current gene name, old gene name, new classification scheme (Group for α-tubulin, Class for β-tubulins), C-terminal sequences of each, and antibodies that recognize those sequences if known. Click here for file

Additional file 4 **KEGG Pathways represented by genes in Additional Table 1**. Data entered into the KEGG Pathways website  yielded a number of pathway hits. Shown here are the top 4 pathways and the genes represented in each. Click here for file

Additional file 5 **GO Terms represented by genes in Additional file **2. Data entered into KEGG Spider  used to generate the pie chart in Figure 3. Click here for file
