# Supplementary material for: Gene expression profiles in mouse embryo fibroblasts lacking stathmin, a microtubule regulatory protein, reveal changes in the expression of genes contributing to cell motility
Source: BMC Genomics. 2009 Jul 30;10:343. doi: 10.1186/1471-2164-10-343 (PMC2725145; doi:10.1186/1471-2164-10-343)
Supplement: Additional file 1 — Primers designed for stathmin family and tubulin isotype qRT-PCR. All primers were designed as described in Methods. Bold nucleotides represent exon junctions in mRNAs. [file 1471-2164-10-343-S1.pdf]

Mouse Gene Specificity	Accession No.		Oligonucleotide Sequence	Amplicon Length, bp	Tm, ºC
GAPDH	NM_008084	Sense:	5'-AGGTCGGTGTGAACG**GA**TTT-3'	80	83
		Antisense:	5'-AACAATCTCCACTTTGCCACTG-3'		
Op18-2/3	NM_019641	Sense:	5'-GTTCGACATGGCATCTTCT**GA**T-3'	70	81
		Antisense:	5'-CTCAAAAGCCTGGCCTGAA-3'		
Op18-4/5	NM_019641	Sense:	5'-AA**GG**ACAAGCACGTGGAAGAG-3'	74	81
		Antisense:	5'-TAGTCAGCCTCAGTCTCATCCG-3'		
α-Tb-1a	NM_011653	Sense:	5'-T**GC**GTGAGTGCATCTCCAT-3'	60	83
		Antisense:	5'-ATTGCCGATCTGGACACCA-3'		
α-Tb-1b	NM_011654	Sense:	5'-ATC**GA**TGAAGTTCGCACCG-3'	60	83
		Antisense:	5'-TGTGATGAGCTGCTCAGGATG-3'		
α-Tb-1c	NM_009448	Sense:	5'-TCTTCAGTGAGACAGGAGCTGG-3'	87	84
		Antisense:	5'-CGGTGCGAACTTCA**TC**GAT-3'		
α-Tb-4a	NM_009447	Sense:	5'-ATC**GA**TGAGATCCGAAATGGC-3'	77	81
		Antisense:	5'-GCTGCATCTTCTTTCCCAGTGA-3'		
α-Tb-3a	NM_009446	Sense:	5'-CGGCAAAGAGATTGTCGACCT-3'	52	82
		Antisense:	5'-ATCGG**CC**AGCTTTCGGATT-3'		
α-Tb-3b	NM_009449	Sense:	5'-TGCCCGACTGGATTTAA**GG**TG-3'	102	83
		Antisense:	5'-ATTGCTCAGCATGCACACAGC-3'		
α-Tb-8	NM_017379	Sense:	5'-GCTGCCATCAAGACCAAGAGAA-3'	76	80
		Antisense:	5'-GGTAGTTGATGCCCACC**TT**GAA-3'		
α-Tb-l3	NM_001033879	Sense:	5'-GGCACCTGCTGATCATCAT**GA**-3'	64	81
		Antisense:	5'-ATCTGGACACCAGCTTGACCA-3'		
β-Tb-I	NM_011655	Sense:	5'-TCAGGTCCTTTTGGCCAGAT-3'	56	80
		Antisense:	5'-CCAGACTGA**CC**GAAAACGAAG-3'		
β-Tb-II	NM_009450	Sense:	5'-AGATCGGCGCTAA**GT**TTTGG-3'	79	82
		Antisense:	5'-TCACTGTCGCCATGGTAACTG-3'		
β-Tb-II	NM_023716	Sense:	5'-CCAGATCGGTGCCAA**GT**TTT-3'	87	80
		Antisense:	5'-TGCAAATCACTGTCTCCATGG-3'		
β-Tb-III	NM_023279	Sense:	5'-CCTGGAACCATGGACAGTGTT-3'	85	82
		Antisense:	5'-CAGCA**CC**ACTCTGACCAAAGA-3'		
β-Tb-IVa	NM_009451	Sense:	5'-GCCCTTTTGGCCAGATCTTTC-3'	56	80
		Antisense:	5'-TGCTCCGGATTGA**CC**AAATAC-3'		
β-Tb-IVb	NM_146116	Sense:	5'-CACTTACCACGGAGATAGCGAC-3'	67	83
		Antisense:	5'-A**CC**GGTGGCTTCGTTGTAGTA-3'		
β-Tb-V	NM_026473	Sense:	5'-GGAACCAGATCGGTACCAA**GT**T-3'	51	80
		Antisense:	5'-TGCCGTGCTCATCACTGAT-3'		
β-Tb-VI	NM_001080971	Sense:	5'-AACCAGATCGGAGCCAA**GT**TC-3'	59	82
		Antisense:	5'-GCGCAGTCAATCCCATGTT-3'		
