# Supplementary material for: Gene expression profiles in mouse embryo fibroblasts lacking stathmin, a microtubule regulatory protein, reveal changes in the expression of genes contributing to cell motility
Source: BMC Genomics. 2009 Jul 30;10:343. doi: 10.1186/1471-2164-10-343 (PMC2725145; doi:10.1186/1471-2164-10-343)
Supplement: Additional file 4 — KEGG Pathways represented by genes in Additional Table 1. Data entered into the KEGG Pathways website yielded a number of pathway hits. Shown here are the top 4 pathways and the genes represented in each. [file 1471-2164-10-343-S4.pdf]

Additional Table 4 – KEGG Pathways represented by genes in Additional Table 1.

Focal adhesion

- Itga9; integrin alpha 9
- Col2a1; collagen, type II, alpha 1
- Col3a1; collagen, type III, alpha 1
- Col1a1; collagen, type I, alpha 1
- Hgf; hepatocyte growth factor
- Itga3; integrin alpha 3
- Itga6; integrin alpha 6
- Itgb7; integrin beta 7
- Lama4; laminin, alpha 4
- Myl7; myosin, light polypeptide 7, regulatory
- Pdgfrb; platelet derived growth factor receptor, beta polypeptide
- Thbs2; thrombospondin 2
- Thbs4; thrombospondin 4
- Itga8; integrin alpha 8
- Itga11; integrin alpha 11
- Tnn; tenascin N
- Col5a3; collagen, type V, alpha 3
- Pik3cb; phosphatidylinositol 3-kinase, catalytic, beta polypeptide

Cell Communication

- Krt5; keratin 5
- Col17a1; collagen, type XVII, alpha 1
- Col2a1; collagen, type II, alpha 1
- Col3a1; collagen, type III, alpha 1
- Col1a1; collagen, type I, alpha 1
- Gjb2; gap junction protein, beta 2
- Gjb4; gap junction protein, beta 4
- Itga6; integrin alpha 6
- Krt17; keratin 17
- Krt19; keratin 19
- Lama4; laminin, alpha 4
- Thbs2; thrombospondin 2
- Thbs4; thrombospondin 4
- Tnn; tenascin N
- Col5a3; collagen, type V, alpha 3

ECM-receptor interaction

- Itga9; integrin alpha 9
- Col2a1; collagen, type II, alpha 1
- Col3a1; collagen, type III, alpha 1
- Col1a1; collagen, type I, alpha 1
- Itga3; integrin alpha 3
- Itga6; integrin alpha 6
- Itgb7; integrin beta 7
- Lama4; laminin, alpha 4
- Thbs2; thrombospondin 2
- Thbs4; thrombospondin 4
- Itga8; integrin alpha 8
- Itga11; integrin alpha 11
- Tnn; tenascin N
- Col5a3; collagen, type V, alpha 3
- Fndc1; fibronectin type III domain containing 1

Regulation of actin cytoskeleton

- Itga9; integrin alpha 9
- Fgf5; fibroblast growth factor 5
- Itga3; integrin alpha 3
- Itga6; integrin alpha 6
- Itgb7; integrin beta 7
- Myl7; myosin, light polypeptide 7, regulatory
- Pdgfrb; platelet derived growth factor receptor, beta polypeptide
- Itga8; integrin alpha 8
- Itga11; integrin alpha 11
- Fgf21; fibroblast growth factor 21
- Myh14; myosin, heavy polypeptide
- Pik3cb; phosphatidylinositol 3-kinase, catalytic, beta polypeptid
